# Case Report: Long-term maintenance of GnRH-a combined with dienogest for thoracic endometriosis syndrome

**DOI:** 10.3389/fmed.2025.1670495

**Published:** 2025-10-27

**Authors:** Huimin Tang, Qiucheng Jia, Wanying Chen, Weiwei Wei, Zongli Ma, Jiming Chen

**Affiliations:** ^1^Department of Obstetrics and Gynecology, The Affiliated Suqian First People’s Hospital of Nanjing Medical University, Suqian, Jiangsu, China; ^2^Department of Obstetrics and Gynecology, The Affiliated Taizhou People’s Hospital of Nanjing Medical University, Taizhou, Jiangsu, China; ^3^Department of Obstetrics and Gynecology, The Second People’s Hospital of Changzhou, the Third Affiliated Hospital of Nanjing Medical University, Changzhou, Jiangsu, China; ^4^Department of Obstetrics and Gynecology, Rugao People’s Hospital, Affiliated Rugao Hospital of Nantong University Xinglin College, Rugao, Jiangsu, China

**Keywords:** thoracic endometriosis syndrome, cyclic haemoptysis, conservative treatment, dienogest, long-term management

## Abstract

Thoracic endometriosis syndrome is a rare form of endometriosis in which endometrial tissue is thought to migrate to the lungs and, with the onset of menstruation, the ectopic foci bleed, causing a range of pulmonary manifestations. In this paper we have collected the clinical data of two patients with thoracic endometriosis syndrome, both of whom presented with haemoptysis as the first symptom and had a history of endometriosis. After 6 courses of treatment with gonadotropin-releasing hormone analogues followed by maintenance treatment with dienogest, the patients’ haemoptysis symptoms were relieved, the CT lesions in the lungs were significantly resorbed compared with the previous ones, and no recurrence was observed at long-term follow-up.

## Background

Endometriosis is a common benign gynaecological condition with a prevalence of up to 10% in women of reproductive age (1). It is characterised by the presence of endometrial tissue in areas other than the uterine cavity and myometrium, usually on the ovaries, bowel or peritoneal surfaces, and in a few cases may involve extra-pelvic organs such as the lungs, liver and ureters. Thoracic endometriosis syndrome (TES) is one of the rarer forms of endometriosis, and about 53–84% of patients with TES have coexisting pelvic endometriosis (2). The most common clinical manifestations of thoracic endometriosis are menstrual pneumothorax (affecting 72–73% of cases), menstrual haemothorax (affecting 12–14% of cases), menstrual haemoptysis (affecting 7–12% of cases) and pulmonary nodules (affecting 2–6% of cases) (3). The first manifestation is usually haemoptysis or chest pain during menstruation, which occurs at the onset of menstruation and resolves with the end of menstruation, with or without chronic cough, menstrual haemothorax, menstrual pneumothorax, recurrent low-grade fever or asymptomatic pulmonary nodules.

There are still no standardised guidelines for the diagnosis and treatment of TES. It is usually treated with conservative pharmacological treatment or surgery. Conservative pharmacological treatment, such as GnRH-a, is rarely used as a primary treatment for TES due to its high side effects and high recurrence rate. For patients with large lung lesions who cannot tolerate surgery or do not wish to undergo surgery, it is particularly important to find a highly effective drug therapy with low side effects. In this paper, we present two patients with TES who were not treated surgically in our hospital, and after maintenance treatment with a highly effective gestagen, dienogest, their clinical manifestations disappeared and their lung lesions were significantly reabsorbed compared with those before treatment, without any adverse effects.

## Case report

### Case 1

Patient A, 27 years old, presented to the outpatient department of Changzhou Second People’s Hospital, affiliated to Nanjing Medical University, on 6 September 2021 with haemoptysis of 5 months’ duration.

The patient had experienced haemoptysis for 5 months without any apparent cause, with a bright red colour and a volume of approximately 5–10 mL/d. The haemoptysis was apparently correlated with the menstrual cycle, occurring 1 day before or 2 days after menstruation, and ceasing after menstruation. The patient had no fever, night sweats, fatigue, weight loss or other symptoms. She had a negative PPD test in a foreign hospital, laryngoscopy: capillary haemorrhage in the larynx and congestion of the mucosa, and antibiotic treatment was ineffective. The patient had a history of one previous pregnancy and one previous delivery, and underwent caesarean section on 8/1/2020; she underwent surgery for abdominal wall endometriosis in our hospital on 9/18/21; there was no history of tuberculosis or exposure, and there was no history of chronic cough, sputum, or haemoptysis.

**Figure 1:**
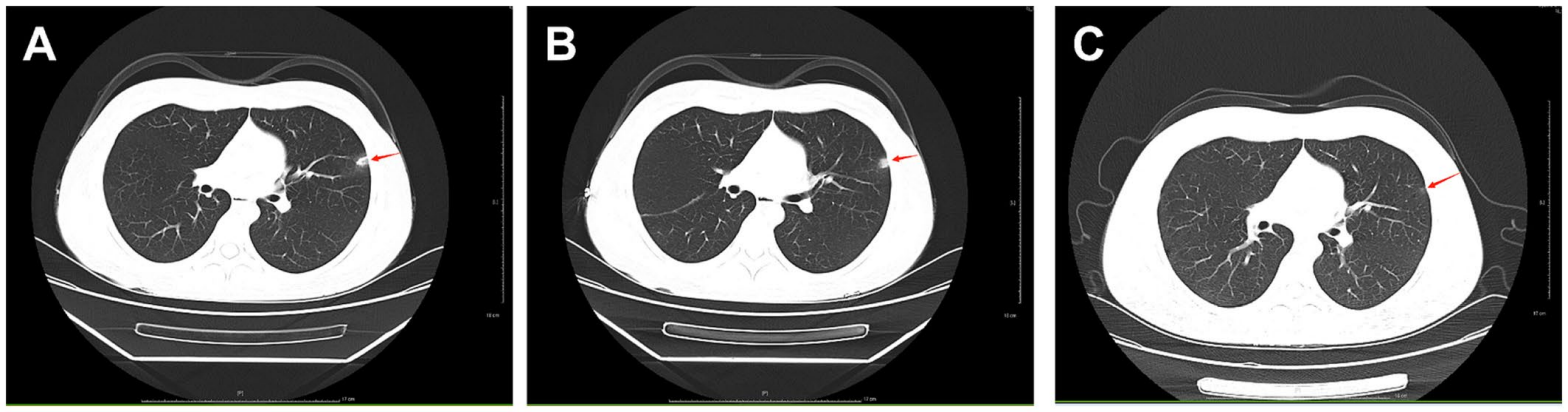
**A**: lungs CT (2021-10-09): nodular slightly dense shadow with maximum diameter of about 0.5 cm in the upper lobe of the left lung. **B**: lung CT (2022-01-04): nodular slightly high-density shadow in the upper lobe of the left lung with a maximum diameter of about 0.5 cm. **C**: lung CT (2023-03-07) with a maximum diameter of about 0.3 cm (the lesion was reduced).

The patient was admitted to hospital on 2021-10-09 for routine blood tests, C-reactive protein, liver and kidney function, and coagulation results showed no obvious abnormalities. CT of the lungs showed: bilateral thoracic symmetry, increased lung texture in both lungs, nodular dense ground-glass opacity in the upper lobe of the left lung with a maximum diameter of approximately 0.5 cm, and striated ground-glass opacity in the lower lobe of the left lung (Figure 1A). Based on the patient’s history and imaging findings, thoracic endometriosis syndrome was suspected, and the gonadotropin-releasing hormone agonist analogue trenbolone acetate 3.75 mg (trade name: Dabigatran) was administered subcutaneously from 16 October 2021 (1 time/1 month, 6 times in total) until 10 March 2022, in combination with oral tibolone tablets 2.5 mg. Thereafter, denogestrel 2 mg qd maintenance was given (March 2022-present). During this period, the patient came to the hospital regularly for physical examination, blood routine, coagulation routine (see Table 1), liver and kidney function (see Table 2), and breast ultrasound, which showed no obvious abnormality. The patient recovered well during the medication period, and no coughing up of blood was observed during the follow-up period, and no new lesions were seen on lung CT, and the diameter of the old lesions decreased compared to the previous ones (see Figures 1B,C). During this period (follow-up to date has been approximately 4 years), the patient had no menstrual flow, no symptoms such as hot flushes and excessive sweating, irritability and insomnia, bone and joint pain, dizziness and headaches.

**Table 1:** Patient A haematology and coagulation routine.

Inspection items	2021-9-8	2022-04-12	2023-03-11
White blood cell count (10^9^/L)	4.38	4.87	6.36
Red blood cell count (10^12^/L)	4.43	4.41	4.24
Haemoglobin (g/L)	119	121	129
Blood platelet count (10^9^/L)	305	305	303
Prothrombin time (s)	11.6	11.7	12.5
Activated partial thromboplastin time (s)	30.6	30.4	30.0
Prothrombin time (s)	18.4	17.8	16.3
Fibrinogen (g/L)	3.02	4.01	4.86

**Table 2:** Liver and renal function, blood lipids in patient A.

Inspection items	2021-9-8	2022-04-12	2023-03-11
Total bilirubin (μmol/L)	10.0	12.7	13.3
ALT (U/L)	14.3	12.5	11.6
AST (U/L)	–	19.7	17.2
ALT/AST	–	0.635	0.674
GLU (mmol/L)	4.78	4.98	5.44
Creatinine (μmol/L)	48.1	48.7	50.2
Uric acid (μmol/L)	263.1	274.7	280.0
Total cholesterol (mmol/L)	–	4.8	4.9
Triglyceride (mmol/L)	–	1.17	1.13

### Case 2

Patient B, 46 years old, was admitted to Changzhou Second People’s Hospital, affiliated to Nanjing Medical University, on 14 September 2020 for “recurrent chest pain and coughing up blood for half a year.”

**Figure 2:**
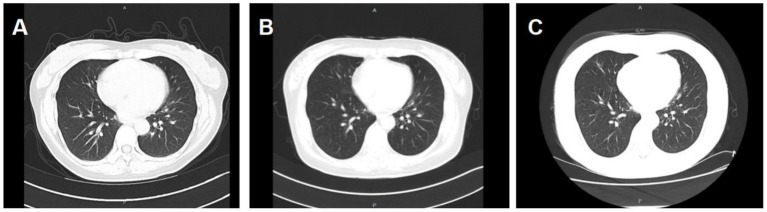
**A**: lung CT (plain scan + enhancement, 2021-01-06): fibrous streaks seen in the middle lobe of the right lung and the lower lingual segment of the upper lobe of the left lung, with well defined borders, and enhancement partially visible on enhanced scan. **B**: lung CT (plain + enhanced, 2022-08-30): striated hyperdense shadows seen in the upper and middle lobes of the right lung and the lower lobe of the left lung, enhancement did not show significant enhancement. **C**: CT lungs (plain scan, 2023-10-10): striated hyperdense shadows seen in the upper and middle lobes of the right lung and the lower lobe of the left.

Six months ago, the patient developed menstrual chest tightness, chest pain, dyspnoea, cough with a small amount of bloody sputum, about 3–5 mL/d. There were no symptoms of fever, night sweats, fatigue, weight loss, etc., and the PPD test was negative at the outpatient clinic. Vaginal ultrasound suggested adenomyosis with fibroids. Lung CT: Lung infection is possible. Anti-infective treatment was given with poor results. Later, several follow-up CT scans showed periodic enlargement and shrinkage of the lung lesions. Combined with the patient’s medical history and imaging findings, thoracic endometriosis syndrome was considered. Goserelin extended-release implant (trade name: Norethindr 3.6 mg) was given subcutaneously from 24 September 2020 (1 time/1 month, a total of 6 times) until 22 March 2021, combined with tibolone tablets 2.5 mg orally, and then denogestrel 2 mg qd for maintenance (March 2021-present). During this period, the patient came to the hospital regularly for follow-up examinations of coagulation routine, blood routine (see Table 3), liver and kidney functions (see Table 4), sex hormones (see Table 5), and breast ultrasound, all of which showed no significant abnormalities. There was no progression of lesions on lung CT (see Figure 2). During the medication period, the patient had no symptoms of coughing up blood and no menstrual flow. She had occasional hot flushes, excessive sweating, irritability and insomnia. A perimenopausal syndrome was considered and she was given Shannon granules, which greatly relieved her symptoms. There were no symptoms of dizziness, breast discomfort, depression, acne, osteoarthralgia, etc. during the medication (follow-up to date has been approximately 5 years).

**Table 3:** Patient B haematology and coagulation routine.

Inspection items	2021-06-22	2021-12-14	2022-04-19	2022-08-30	2023-01-31	2023-10-10
White blood cell count (10^9^/L)	4.16	5.35	4.55	5.59	5.42	6.13
Red blood cell count (10^12^/L)	4.65	4.76	4.85	4.82	4.71	4.76
Haemoglobin (g/L)	144	151	151	151	148	152
Blood platelet count (10^9^/L)	244	269	259	246	240	258
Prothrombin time (s)	11.5	10.9	11.4	10.4	11.6	10.9
Activated partial thromboplastin time (s)	28.2	29.2	28.2	27.4	28.6	26.9
Prothrombin time (s)	18.6	18.8	19.1	18.3	18.0	15.5
Fibrinogen (g/L)	2.28	2.6	2.86	2.77	1.69	2.89

**Table 4:** Liver and renal function, blood lipids in patient B.

Inspection items	2021-06-22	2021-12-14	2022-04-19	2022-08-30	2023-01-31	2023-10-10
Total bilirubin (μmol/L)	15.1	12.4	18.2	13.8	15.8	12.0
ALT (U/L)	13.0	14.3	23.0	41.4	17.1	46.6
AST (U/L)	14.3	15.7	18.4	31.9	16.1	30.8
ALT/AST	0.91	0.91	1.25	1.298	1.062	1.513
GLU (mmol/L)	5.24	5.61	5.64	5.54	4.96	5.34
Creatinine (μmol/L)	65.3	61.6	61.5	59.3	57.7	66.4
Uric acid (μmol/L)	222.3	231.1	217.8	282.1	220	264
Total cholesterol (mmol/L)	3.75	3.79	3.98	3.78	4.04	3.25
Triglyceride (mmol/L)	1.47	1.57	2.09	1.74	1.74	2.42

**Table 5:** Sex hormone levels in patient.

Inspection items	2021-01-04	2021-06-22	2021-12-14	2022-04-19	2022-08-30	2023-01-31
E2 (pmol/L)	75.76	118.6	239.5	159.6	108.2	413.6
Androstenedione (nmol/L)	0.95	1.43	1.55	1.31	0.83	0.77
Progesterone (nmol/L)	0.3	1.03	0.95	0.53	0.4	0.7
Lactogen (ng/ml)	9.44	25.2	28.95	24.6	26.08	17.87
Luteinising hormone (mIU/ml)	0.29	13.51	3.94	2.1	2.27	3.2
Follicle stimulating hormone (mIU/ml)	4.63	26.24	8.25	6.93	4.84	4.9

## Discussion

There is still uncertainty about the pathogenesis of TES and the most clinically convincing theory at present is lymphatic and haematogenous spread. Researchers believe that endometrial tissue metastasises from the pelvis to the lungs or other sites via vascular/lymphatic channels. Some autopsies of patients with thoracic endometriosis have shown that lesions are more common in the bronchi bilaterally, whereas the diaphragm is more common on the right side, which may be the reason why the right side of the diaphragm is more rich in lymphatic vessels (4). The theory also fully explains why the endometrium is planted in distant parts of the body.

Diagnosis of TES mainly involves imaging and pathological diagnosis. Laboratory tests are less commonly used for clinical diagnosis, although some reports have shown that CA125 levels are elevated in patients with TES, which has some value in diagnosing the disease. However, its lack of specificity is poor and the level changes with the menstrual cycle, so it is usually used as a reference indicator (5). The imaging manifestations of TES include intrapulmonary lamellar opacities, nodular opacities, ground-glass opacities, opacities, pleural effusion, pneumothorax, and the lesions mostly involve unilateral lungs, but can also be seen in both lungs, but the above changes have no specificity. As the lesions are related to the menstrual cycle, CT scanning of the chest can be dynamically observed. CT scans during menstruation show obvious exudation or enlarged changes, and after menstruation the lesions can be seen to shrink by themselves or to disappear for the most part. However, CT radiation is high and MRI is gradually being used for the clinical diagnosis of TES because of its better resolution and better localisation of pulmonary haemorrhage through multiple sequences. There is a gold standard for the diagnosis of TES that relies heavily on pathology. The typical “trinity” of endometrial glands, mesenchyme and ferritin-containing macrophages is seen in only one third of surgical specimens. Therefore, if no endometrial glands are present in a tissue sample or only a small piece of endometrial mesenchyme is found, the use of immunohistochemical methods using antibodies against ER, PR and CD10 can also aid in the diagnosis (6).

As both patients in this paper declined surgery, pathological diagnosis was not performed. We therefore conducted a comprehensive and systematic differential diagnosis. Infectious diseases, such as tuberculosis, are the most common cause of haemoptysis. However, both patients resided in regions with low tuberculosis prevalence, and their PPD tests were negative. Imaging studies revealed no characteristic features of pulmonary tuberculosis, such as tree-in-bud signs, miliary nodules or calcified cavities. Furthermore, neither patient exhibited any of the typical symptoms of tuberculosis, such as low-grade fever, night sweats or significant weight loss. Consequently, tuberculosis was initially ruled out. Regarding other pulmonary infectious diseases, such as lung abscess or bronchiectasis, imaging studies, symptoms and ancillary tests provided no supporting evidence. Concurrently, imaging studies excluded non-infectious pulmonary conditions, including malignant lung tumours and vascular malformations. Regarding other potential haemorrhagic lesions, coagulation disorders were ruled out through platelet and coagulation function testing. Given the patient’s haemoptysis occurring in relation to her menstrual cycle, we considered a possible association with TES. Following treatment with the hormonal agent GnRH-a, the haemoptysis gradually resolved. This therapeutic response indirectly confirmed the lesion’s hormone dependency, providing strong clinical evidence for the diagnosis of TES.

Treatment of TES is aimed at suppressing endometrial tissue and preventing further seeding. Pharmacological and surgical treatments are currently the most clinically effective treatment modalities. The treatment plan should be individualised according to the patient’s age, fertility aspirations, lesion size and location, and symptoms. Surgical treatment is more appropriate for some peripheral pulmonary nodular TES confined to a single lobe, and surgical resection is more traumatic and has a greater impact on lung function for some lesions that are widespread and not confined to a single lobe. Large-scale evidence supports the role of postoperative hormonal therapies (e.g., DNG, OC, GnRH agonists/antagonists) in reducing recurrence in thoracic endometriosis-related pneumothorax (TERP), a major component of TES (7). While TERP and the present TES cases differ, such data provide contextual support for hormonal strategies in thoracic manifestations of endometriosis. The main principle of conservative pharmacological treatment is to inhibit ovarian oestrogen secretion, including GnRH-a, danazol, oral contraceptives and progestogens. Gonadotropin-releasing hormone minimises the secretion of follicle-stimulating hormone and luteinising hormone, resulting in hypogonadism. Treatment with GnRH-a for 6 months resulted in complete resolution of endometriotic foci and effusions in half of the patients (8). The main side effects are due to perimenopausal symptoms caused by hypoestrogenaemia, such as hot flushes, vaginal dryness, decreased libido, insomnia and depression. Long-term use is associated with bone loss (9). Danazol and oral contraceptives inhibit the pituitary-ovarian axis and reduce estrogen production, and their adverse effects are more common, mainly weight gain, fluid retention, fatigue, acne, oily skin, facial flushing and emotional instability, and long-term use produces a greater burden on the patient’s body (10). Therefore, a highly effective progestogen with fewer adverse effects in long-term treatment should be sought for the pharmacological conservative management of TES patients. As the fourth generation of synthetic potent progestins, dinogestrel has gradually entered the clinical picture.

Denogestrel is a derivative of 19-nortestosterone, which has been shown to bind highly selectively to the progesterone receptor with moderate estrogenic inhibition, and has strong antiproliferative, antiangiogenic and anti-inflammatory properties on endometriosis implants, while having some antiandrogenic activity, but no significant estrogenic, glucocorticoid or salicorticoid activity, and little effect on metabolic parameters (11). The new progestin denogestrel has both central and peripheral mechanisms of action, relieving endometriotic dysmenorrhoea while reducing endometriotic foci, with the reduction in foci being more pronounced with prolonged dosing. This drug has fewer adverse effects, with a small number of patients experiencing headache and breast discomfort, and with less estrogen suppression, symptoms such as osteoporosis and insomnia are more readily accepted (12). Dinogestrel has now been shown to be highly effective in relieving pain, inhibiting lesions, etc., as well as being safe, well tolerated and with few adverse effects, and has gradually become an effective drug for the long-term management of patients with endometriosis. And because dienogest’s high progestational effect makes it more easily absorbed, it is gradually being used to treat patients with endometriosis at more distant sites (13, 14). Why was the combined strategy of ‘GnRH-a followed by dienogest’ adopted for the two patients discussed in this paper? This approach combines the advantages of both medications perfectly. GnRH-a provides rapid disease control, while dienogest offers high efficacy and long-term safety. The aim is to induce prompt clinical remission, followed by the most suitable medication for sustained maintenance to prevent recurrence. For challenging conditions such as thoracic endometriosis, it represents an aggressive and persistent therapeutic strategy.

Currently, long-term conservative treatment of TES patients with clinical drugs alone, such as oral contraceptive pills and GnRH-a, has a low success rate, with a proven recurrence rate of 50% and its side effects (15). We present two cases of TES patients who were collected for drug management over a three-year period and treated with long-term conservative treatment with dienogest, both of whom showed significant improvement in their symptoms. There were no significant adverse events during follow-up and lung CT lesions gradually decreased. In the clinical literature on TES, there are only three cases of TES treated with dinogestrel (see Table 6) (16, 17). We report the diagnosis and treatment here to provide experience for future clinical diagnosis and treatment of this type of disease.

**Table 6:** Cases of TES treated with dienogest.

Case	Gender (age)	Symptom	Chest CT findings	Treatment	Dosage	Follow up	Recurrence
Leonardo-Pinto et al. (16)	23	Coughing up blood during menstruation, accompanied by pain in the right shoulder	Frosted glass haze area in the upper lobe of the right lung	Dienogest	2 mg QD	1 year	Yes
Hwang et al. (17)	25	Coughing up blood during menstruation	grinded glass image of the right middle lobe	GnRH-a + dienogest	GnRH-a*3 months + dienogest 2 mg QD	10 months	Yes
Hwang et al. (17)	35	Coughing up blood during menstruation	Ground-glass shadow in the right upper lobe	GnRH-a + dienogest	GnRH-a*3 months + dienogest 2 mg QD	49 months	Yes

## Data Availability

The original contributions presented in the study are included in the article/supplementary material, further inquiries can be directed to the corresponding authors.
